# The Flail and Pulseless Upper Limb: an Extreme Case of Traumatic Scapulo-thoracic Dissociation

**DOI:** 10.5704/MOJ.1507.002

**Published:** 2015-07

**Authors:** SW Maria, J Sapuan, S Abdullah

**Affiliations:** Department of Orthopaedics, Faculty of Medicine, Universiti Kebangsaan Malaysia, Cheras, Malaysia

**Keywords:** Scapulo-thoracic dissociation, complete brachial plexus injury, subclavian artery injury

## Abstract

Scapulo-thoracic dissociation is an infrequent injury resulting from high energy trauma which is often associated with severe neurological and vascular injuries which may be unrecognised at the time of presentation. A 24 year-old female presented with bilateral rib fractures, pneumothorax, liver and kidney injuries following a road traffic accident. She also sustained fractures of her right scapula, odontoid, right transverse processes of the thoracic and lumbar vertebrae and a closed fracture of her right femur. Her right upper limb was later noted to be flail and pulseless, due to complete right brachial plexus injury, scapula-thoracic dissociation and subclavian artery avulsion. We managed the upper limb injuries non-operatively, and focused on resuscitation of the patient. Early exploration of the complete brachial plexus injury was not undertaken in spite of the possible associated poor functional outcome as there was no life-threatening indication.

## Introduction

Scapulo-thoracic dissociation is a traumatic lateral displacement of the scapula, which has been described as a closed traumatic forequarter amputation^1^. It is a high energy trauma and is associated with 10% mortality^2^. Associated injuries include vascular and nerve injury. Fractures of the shoulder girdle and dislocations of sterno-clavicular and acromio-clavicular joints can occur. The most common long term result is complete loss of motor and sensory function of the upper extremity. Neurological involvement is the most significant factor in predicting functional outcome^3^.

## Case Report

**Fig. 1:**
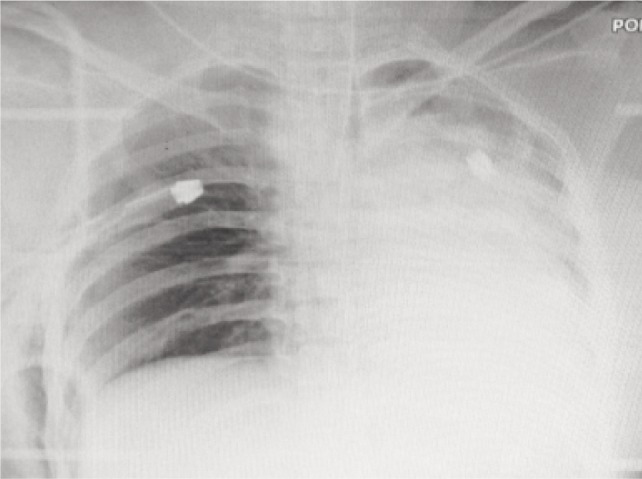
Chest radiograph with ribs fractures and pneumothorax.

A 24-year old female presented to the accident and emergency department following a high energy motor vehicle accident in which she was dragged off her motorbike by her right upper limb by a truck. She was brought in with a Glasgow Coma Scale (GCS) of 8/15, tachycardic, hypotensive and tachypneic. The initial chest radiograph showed bilateral pneumothorax and chest tubes were inserted (Figure 1). She was intubated and appropriately resuscitated and later transferred to the intensive care unit.

**Fig. 2:**
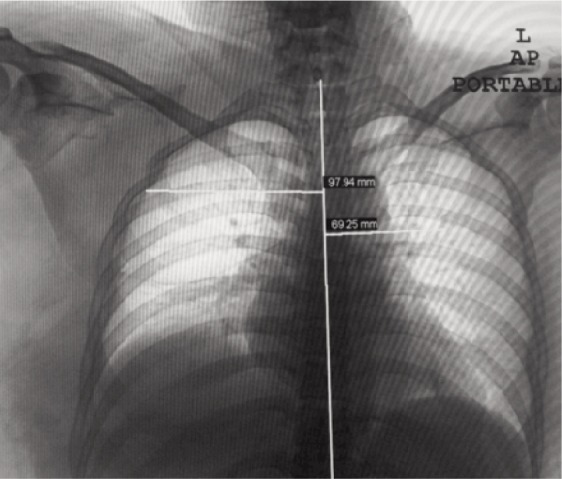
Scapulothoracic dissociation, scapula fracture, ACJ separation. Scapula index: 1.41 (97.94/69.25). Normal average index is 1.07. Scapulothoracic dissociation is suspected if the ratio is 1.29 and above.

**Fig. 3:**
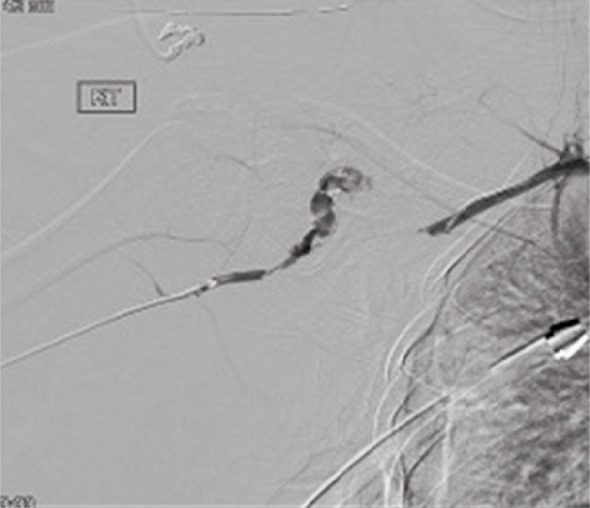
CTA showing subclavian artery long segment (60mm) avulsion with contrast extravasation. Minimal collaterals noted with reconstitution of the distal axillary artery.

CT cervical spine revealed an odontoid fracture. She had bruising and gross swelling over her right shoulder and scapula and closed fracture of her right femur. She had a right scapula fracture with scapula-thoracic dissociation and acromioclavicular disruption. (Figure 2). She also had right transverse process fractures of T4-6, T12, and L1-4. Her abdomen revealed tenderness and guarding over the right quadrant. CT abdomen revealed grade 3 liver and kidney injuries Both the liver and kidney injuries were treated conservatively and she was appropriately transfused. It was noted that there was no movement of her right upper limb and peripheral pulses were absent despite having a warm hand with good capillary refill time. An urgent computed tomography angiography revealed subclavian artery avulsion (Figure 3).

This vascular injury was treated conservatively as she had good hand perfusion from collateral blood supply with no evidence of on-going exsanguination. As her general condition improved, she was gradually weaned off the ventilator and chest tubes and her right upper limb neurology was re-assessed. She had a postganglionic supraclavicular complete brachial plexus injury. After a week, she subsequently underwent internal fixation for her right femur and halo vest immobilisation for odontoid fracture. The scapula fracture and the scapulothoracic dissociation were treated conservatively and she was put on arm sling. The complete brachial plexus injury was approached conservatively and she was started on physiotherapy.

## Discussion

Scapulo-thoracic dissociation is a rare injury resulting from high energy trauma involving lateral traction of the upper extremity or direct blunt impact. Masmejaam *et.al.* reported a 10% mortality and 88-100% risk of associated vascular injuries^2^. Such severe traction mechanism has a wide zone of injury involving musculature and neurovascular structures and is associated with significant morbidity. The prevalence of brachial plexus injuries was 94% and usually complete^4^. Kelbel *et.al.* described specific radiographic criteria which is the “scapula index” to diagnose scapula-thoracic dissociation^3^. They measured the distance from the scapula’s medial borders to the spinous process on non-rotated chest radiograph, and these figures were divided to obtain- a ratio, which is normally 1.07 (Figure 2). Zelle *et. al.* reported that scapula index of 1.29 indicated a scapula-thoracic dissociation^4^.

Damschen *et. al.* proposed a simple classification system based on the presence or absence of either a vascular or neurological injury^5^. A Type 1 injury involved an isolated musculoskeletal injury, Type 2 involved a musculoskeletal injury with either a vascular or neurological injury and a Type 3 involved musculoskeletal and both a vascular and neurological component. Despite the fact that Type 2 and 3 injuries would logically represent more devastating and disabling injuries, functional outcome data do not correlate with Damschen’s system of classification.

Zelle *et. al.* found that the completeness of brachial plexus injury was most predictive of patient’s functional outcome. The authors found that although a vascular injury is more life threatening, the patient’s neurological injury was the most significant life-altering morbidity. They proposed a fourth component to the Damschen classification which includes all patients with a complete brachial plexus injuries^4^. Urgent surgical exploration is indicated for patients with active haemorrhage, an expanding hematoma or hand ischaemia. In the event of vascular exploration it is recommended to inspect the trunks of the brachial plexus and if a complete disruption is found, primary amputation should be considered^4^.

We managed our patient’s right upper limb non-operatively in the early stages since the vascular, neurological and bone injuries were not life threatening. Despite the expected poor functional outcome of these conditions, the patients survived the major polytrauma.

We recommend constantly alertness to scapulothoracic dissociation which may present as a component of severe polytrauma. Priority is focused on resuscitation of the patient, and surgery should only be considered when there are definite life threatening indications.
